# Comparison of immunochromatographic diagnostic test with Hheminested Reverse transcriptase polymerase chain reaction for detection of rabies virus from brain samples of various species

**DOI:** 10.14202/vetworld.2015.135-138

**Published:** 2015-02-09

**Authors:** Pranoti Sharma, C. K. Singh, Deepti Narang

**Affiliations:** 1 Department of Veterinary Pathology, Guru Angad Dev Veterinary and Animal Sciences University, Ludhiana - 141 004, Punjab, India; 2 Department of Veterinary Microbiology, Guru Angad Dev Veterinary and Animal Sciences University, Ludhiana - 141 004, Punjab, India

**Keywords:** antigen, heminested, immunochromatographic, rabies

## Abstract

**Aim:**

Detection of rabies is a cause of serious concern in developing countries, where dearth of highly equipped laboratories and trained personnel to handle sophisticated investigations is felt. The availability of a diagnostic kit, which can be used in the field, is essential for diagnosis and control programs as well as for epidemiological surveillance of the prevalence of the disease. This study was planned to evaluate anigen rabies Ag test kit for its efficacy to be used for rapid diagnosis of rabies under field conditions. The test results were compared with hemi-nested reverse transcriptase polymerase chain reaction and with a gold standard fluorescent antibody test.

**Materials and Methods:**

A total of 34 brain samples from different rabies suspected animals including dogs, buffaloes, cow, horse, and cat were examined in this study.

**Results:**

Sensitivity of the kit was found to be 91.66%, specificity 100%, and accuracy was 94.11%.

**Conclusion:**

The study implies that the immunochromatographic diagnostic test kit may be employed for diagnosis of rabies in field conditions.

## Introduction

Rabies is a fatal encephalomyelitis infecting a wide range of animal species and humans. It is caused by *Lyssavirus* of the family Rhabdoviridae [1]. 55,000 persons are expected to die of rabies every year, though it may still be less than the actual figure [2]. In this context, there is always a need to develop and produce rapid and reliable test for rabies, which can be employed in field conditions to evaluate the real impact of this dreaded zoonotic disease. At present, diagnosis of rabies infection requires detection of rabies virus (RABV) by fluorescent antibody test (FAT) in brain samples. World Health Organization (WHO) and World Animal Health Organization (OIE) have recommended FAT as the gold standard test for confirmatory rabies diagnosis.

Despite high sensitivity of FAT in rabies diagnosis, FAT has many drawbacks, which limits its usefulness, including establishment of FAT laboratories requires expensive infrastructure, well-trained technician and getting fresh specimens to potentially distant laboratories in an adequate condition for testing is unfeasible [3-5]. Other laboratory techniques for routine diagnosis *viz*. mouse inoculation test and rabies tissue culture infection test require several hours of processing for obtaining the final result and therefore are not feasible under field conditions. Molecular methods allow the detection of genetic material in a short period of time. Among various molecular approaches, with hemi-nested reverse transcriptase polymerase chain reaction (HnRT-PCR) is considered to be most sensitive [6,7]. Since, molecular approaches like HnRT-PCR also require sophisticated laboratory facilities and highly trained technical staff, thus, there is impending need of a commercially available diagnostic kit that could be employed for efficient diagnosis of rabies in field conditions.

Thus, this study was envisaged to study the efficacy of a commercially available diagnostic immunochromatographic kit for detection of rabies antigen from BioNote Inc. Seoul, Korea in comparison with a sensitive molecular approach of rabies diagnosis *viz*. Hn RT-PCR.

## Materials and Methods

### Ethical approval

The study was approved by Institutional Animal Ethics Committee.

### Species of animals

A total of 34 tissues samples from cerebellum and hippocampus of the brain of different suspected animals *viz*. dogs (n=13), buffaloes (n=11), cows (n=8), horse (n=1) and cat (n=1) were collected from postmortem hall of the Department of Veterinary Pathology as well as from Veterinary Clinics of GADVASU from different districts of Punjab. Samples were stored in −80°C (Ultra-low-temperature freezer, Haier, Biomedical) till processing.

### Immunochromatographic diagnostic test

Test was obtained using immunochromatographic diagnostic test kit *viz*. anigen rabies Ag test BioNote Inc. Seoul, Korea. The immunochromatographic assay employed direct sandwich method wherein 10% homogenate of each brain sample was formed in phosphate buffered saline (PBS). Brain homogenate was collected using a swab and mixed in the diluent tube provided in the test kit. Purple colored band moved across the result window in the center of the test device indicating proper loading of the samples. Results were interpreted within 5-10 min. In order to study the effectiveness of the diagnostic kit, the recommended dilution of test samples of brain tissue i.e. 1:10 was further diluted with 10-fold dilutions in PBS as 1:100, 1:1000, 1:10,000, and 1:100,000.

### Analysis

Test kit revealed positive control band in the left section of the result window and test band in the right section of the window. In positive cases, both positive control color band as well as test sample band appeared, whereas, in negative cases, the test band failed to appear. Absence of positive control band rendered the test invalid.

### HnRT-PCR

About 10% brain homogenate, as prepared for the kit, was subjected for RNA extraction by Trizol (Invitrogen USA), followed by cDNA synthesis using applied biosystems reverse transcriptase kit with RNase inhibitor. cDNA was subjected to PCR amplification using primers targeted towards conserved blocks of RNA-dependent RNA polymerase or large protein (L). Primers PVO5 (10 pmol/µl) and PV09 (10 pmol/µl) were used in primary PCR and primers PV05 and PV08 (10 pmol/µl) were used in HnRT-PCR. Primary PCR was carried out in 25 µl of reaction volume using GoTaq green PCR master mix (Promega) and 2 µl of cDNA, subjected to PCR cycling conditions initial denaturation at 94°C for 3 min amplification for 35 cycles, denaturation at 94°C for 30 s, hybridization at 56°C for 45 s, elongation at 72°C for 40 s and final extension step of 3 min at 72°C with slight modifications as reported earlier [8]. Primary PCR served as a template for secondary with the same PCR cycling conditions.

### FAT

Duplicate impression smears of 1 cm diameter on either end of the labeled slides were prepared from cerebellum/hippocampus of each brain specimen. Control positive slides from known rabies positive case and control negative slide from normal, uninfected and unvaccinated animal was also prepared along the smear. The impression smears were air-dried and fixed in cold acetone overnight. Impression smear slides were air dried and 0.1 ml of anti-rabies nucleocapsid fluorescein isothiocyanate conjugate, (Bio-Rad, France) was added on each smear and incubated at 37°C for 30 min in a moist chamber. The slides were then washed in two successive 0.01 M PBS baths for 5 min each. Thereafter, smears were the air-dried and mounted in 90% glycerine buffer (pH 8.5) and covered with a coverslip. The slides were examined using an AHBT3-RFC reflected light fluorescence attachment (Olympus, Japan).

Sensitivity, accuracy and specificity were calculated as under:


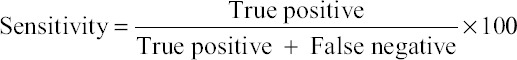



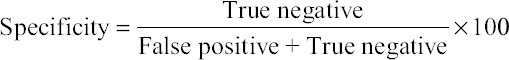



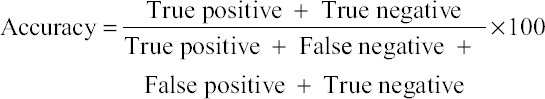


Kappa measure of agreement was determined by using Graph Pad Software Kappa is a measure of agreement that shows whether a test correctly predicts an outcome. The kappa value of agreement levels was interpreted as follows: Poor agreement ≤0.20, fair agreement =0.20-0.40, moderate ­agreement =0.40-0.60, good agreement =0.60-0.80, and very good agreement ≥0.80 [9].

## Results

FAT examination of 34 brain samples established 24 cases as true positive for rabies on the basis of the presence of characteristic apple green fluorescence in positive samples.

### Immunochromatographic diagnostic test

**Figure-1:**
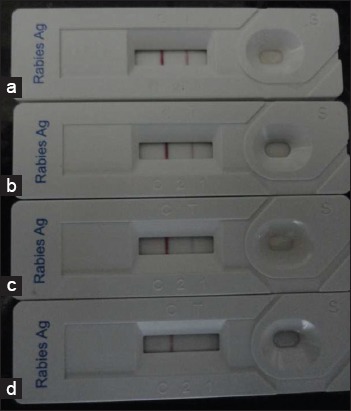
Detection limit immunochromatographic diagnostic test, (a) 1:100 dilution, strong positive bands were observed both in the test line and the control line, (b) 1:1000 dilution, weak positive band was observed in the test line and strong band in control line, (c) 1:10,000 dilution, very weak positive band was observed in test line and strong band in control line, (d) only control band appeared, negative result.

In the present study, of 34 brain samples tested, the control band and test band were revealed in 22 samples therefore 22 samples were found positive with this assay. The diagnostic kit continued to reveal positive reaction up to 1:100,000 dilutions. However, the intensity of color decreased with every 10-fold dilution (Figure-1). Immunochromatographic diagnostic test kit was 91.7% sensitive, 100% specific and 94.11% accurate for rabies diagnosis from brain samples (Table-1). Kappa measure of agreement was 0.86 between immunochromatographic kit and FAT.

**Table-1:** Test results of FAT and immunochromatographic test kit.

Test	Brain FAT positive	Brain FAT negative	Total
Number of animals positive by ICT kit	22	0	22
Number of animals negative by ICT kit	2	10	12
Total	24	10	34

Sensitivity=22/22+2×100, Specificity=10/0+10, Accuracy=22+10/22+0 + 10+2, FAT=Fluorescent antibody test, ICT=Immunochromatographic diagnostic test

### HnRT-PCR

RNA was extracted from 34 brain samples, 260/280 ratio of extracted RNA was in the range of 1.80-1.98. Primary PCR amplification yielded 319 bp products, and secondary PCR yielded 250 bp products. HnRT-PCR targeting L gene diagnosed 24 samples out of the 34 samples as positive, which were ­considered as true positive in the results of FAT examination, thus, indicated 100% agreement with the gold standard FAT (Table-2). Sensitivity of HnRT-PCR was assessed by 10 fold serial dilution of the cDNA and positive results were obtained up to 1 fg.

**Table-2:** Test results of FAT and HnRT-PCR.

Test	Brain FAT positive	Brain FAT negative	Total
Number of animals positive HnRT-PCR	24	0	24
Number of animals negative HnRT-PCR	0	10	10
Total	24	10	34

Sensitivity=24/24+0×100, Specificity=10/0+10, Accuracy=24+10/24+0+10+0, FAT=Fluorescent antibody test, HnRT-PCR=Hemi-nested reverse transcriptase polymerase chain reaction

## Discussion

In developing countries like India, the low level of commitment to rabies control is partly attributable to lack of accurate and extensive surveillance data to indicate the actual disease burden and frequent misdiagnosis of rabies. Limitations of conventional methods and sophistication of molecular approaches calls for an alternative approach. The present study evaluated the efficacy of immunochromatographic kit to be used under field condition for rabies diagnosis and obtained 91.7% sensitivity, which was in complete agreement to the earlier findings [10], when rapid immunodiagnostic test applied on 54 brain samples. However, in other study [11], immunochromatographic kit was evaluated on European mammals and reported sensitivity of 88%, which is comparatively lesser to the findings of the present study. In earlier study on dog brain samples, two types of immunochromatographic diagnostic test targeting different epitopes of viral gene were evaluated and reported sensitivity of 95.5% and 92.3% respectively with Type I and Type II kits [12]. In an effort to develop range of sensitivity, brain samples of various mammals including human were evaluated and range of 74-95% sensitivity, 98-100% specificity and 91-98% accuracy were obtained for various isolates of RABV from different parts of the world [13]. Very good strength of agreement was observed between immunochromatographic kit and FAT and in between HnRT-PCR and FAT.

In the present study, 100% agreement was observed between FAT and HnRT-PCR targeting L gene of virus. Similar conclusions were drawn in other study when one step RT-PCR was analyzed on 68 brain samples in comparison with L gene targeted HnRT-PCR and FAT [14]. There are few studies targeting L gene of RABV in brain tissue using HnRT-PCR, however, there are reports of studies targeting G-L intergenic region of the virus [15]. HnRT-PCR can be used as an alternative approach where fluorescent microscope facility is not available. Moreover, it can also be used when samples are in a decomposed state. In addition, the amplified products can be used in techniques such as sequencing and for epidemiological characterization of the virus [16].

Careful perusal of the literature reveals comparison of immunochromatographic kit and HnRT-PCR has not reported so far for rabies diagnosis. In this significant approach, though, HnRT-PCR was found to be more sensitive and accurate than immunochromatographic kit while detecting RABV from brain samples, but it requires highly equipped laboratories and well-trained personnel to handle this investigation, which is in its infancy in the developing countries. Since rabies is a disease of grave concern and to achieve effective control on the disease rapid and reliable diagnosis is desirable. Immunochromatographic kit can be used for rabies surveillance study and for diagnosis in field condition. As immunochromatographic kit is less time consuming, less hazardous and requires no special equipment and trained personnel to provide results.

## Conclusion

For efficacious diagnosis of rabies in field conditions, immunochromatographic kit used in the present study may be used to serve as an indicative test that is conveniently feasible in the field conditions. However, the samples found negative by the kit may be investigated further by FAT or other molecular approaches for authenticating the diagnosis of rabies.

## Author’s Contributions

CKS and DN designed the experiment, sample collection and Experiment was performed by PS under the supervision of CKS. Manuscript preparation was supervised reviewed and edited by CKS and DN. All authors read and approved the final manuscript.
